# Genome Sequences of Microbacterium foliorum Phages Anseraureola, Pondwater, and Yasuo

**DOI:** 10.1128/mra.00849-22

**Published:** 2022-10-13

**Authors:** David Bamgbowu, John Bsoumai, Jessica Butura, Emma Cady, Gabriella Cholod, Isaac Collibee, Lois Dompreh, Sydney Eisner, Maxim Elmaleh, Katelan Fitzgerald, Eric Gillis, Anna Horgan, Dylan Judd, Julia Keefe, Erika Kovalski, Kylie LaBianca, Patrick Lee, Fengkai Lin, Heather Maiuri, Chloe McDonald, Arden McKnight, Meghan Meseerole, Farah Mizra, Elizabeth Monger, Evelyn Moore, Nina Nguyen, Bettina Noel, Daniel O’Connor, Ruben Pagani, Makenzie Palmgren, Karin Pan, Bondita Pech, Jeanne Qian, Sheida Rastegar, Brittney Simas, Adeline Southard, Matt Tracy, Harry Vuong, Sean Whelan, Angela Zou, Elizabeth Punska, Robert Pause, Fengqiu Zhang, Alexander Ribbe, Peter Chien, Jessica Rocheleau

**Affiliations:** a Department of Biology, University of Massachusetts, Amherst, Massachusetts, USA; b Department of Environmental Science, University of Massachusetts, Amherst, Massachusetts, USA; c Department of Microbiology, University of Massachusetts, Amherst, Massachusetts, USA; d Department of Biochemistry and Molecular Biology, University of Massachusetts, Amherst, Massachusetts, USA; e Department of Veterinary and Animal Sciences, University of Massachusetts, Amherst, Massachusetts, USA; f Department of Polymer Science and Engineering, University of Massachusetts, Amherst, Massachusetts, USA; Portland State University

## Abstract

Anseraureola, Pondwater, and Yasuo are bacteriophages with siphovirus morphology that infect Microbacterium foliorum NRRL B-24224. They were isolated from soil collected in Amherst, Massachusetts, and have genome lengths between 17,362 bp and 17,453 bp. These phages each contain 25 predicted protein-coding genes and are assigned to phage cluster EE.

## ANNOUNCEMENT

**FIG 1:**
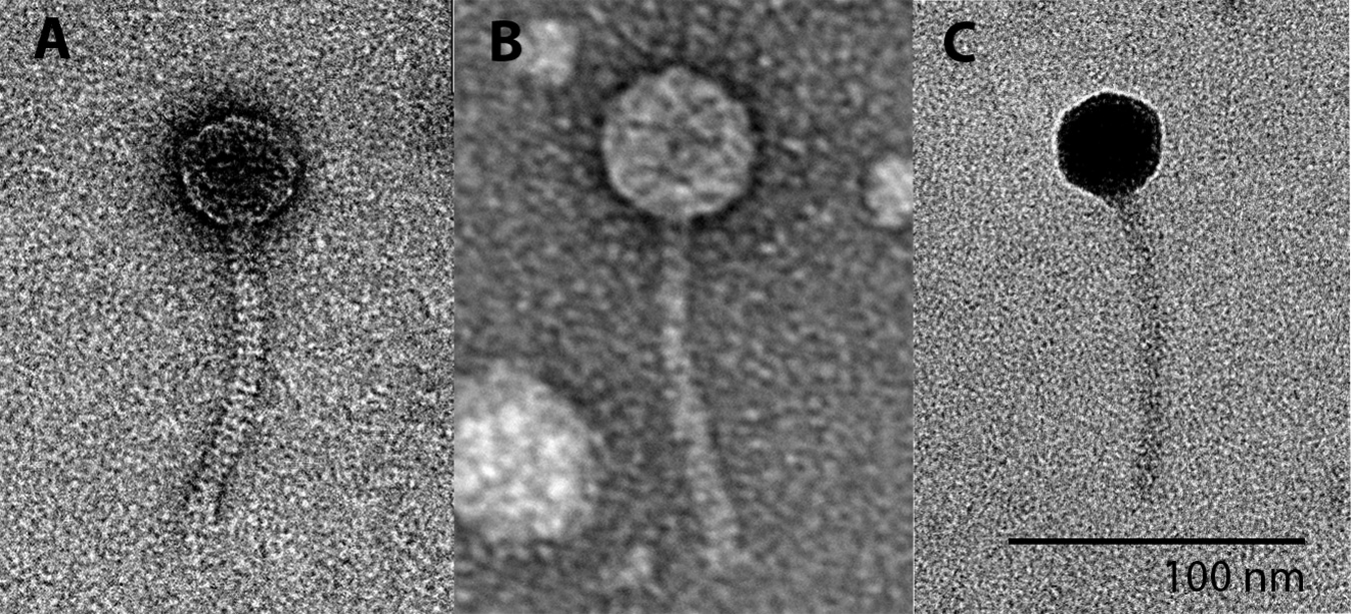
Transmission electron micrographs of phage lysates stained with 1% uranyl acetate, showing *Microbacterium* phages Anseraureola (A), Pondwater (B), and Yasuo (C).

Isolation and sequencing of bacteriophages infecting *Microbacterium* hosts have revealed their genomic diversity (1) and may provide insights into their potential applications in treating *Microbacterium* infections (2) and in biotechnology (3). Here, we report on the bacteriophages Anseraureola, Pondwater, and Yasuo, which were isolated from soil in Amherst, Massachusetts, using Microbacterium foliorum and standard isolation methods (2) (see Table 1 for the sampling location coordinates). Soil samples were suspended in peptone-yeast extract-calcium (PYCa) liquid medium, and the suspension was passed through a 0.22-μm filter. The filtrate was then plated in top agar with Microbacterium foliorum NRRL B-24224 and incubated at 30°C. All three phages were purified with two rounds of plating and produce clear plaques with turbid halos surrounding them, ranging in size from 2 to 4 mm, after incubation at 30°C for 48 h. Negative-staining transmission electron microscopy showed that all three phages have siphovirus morphology (Fig. 1). Yasuo’s capsids measure 35 to 38 nm and tails are 102 to 107 nm, Anseraureola’s capsids are 40 to 42 nm and tails are 105 to 108 nm, and Pondwater’s capsids are 50 to 55 nm and tails are 115 to 117 nm (*n* = 6). All measurements were made using ImageJ v1.53r21 (3).

**TABLE 1:** Genome assembly results for *Microbacterium* phages Anseraureola, Pondwater, and Yasuo

Phage	Sampling location coordinates	Avg sequencing coverage (×)	No. of sequencing reads (thousands)	Cluster	Genome ends	GC content (%)	No. of genes
Anseraureola	42.389642N, 72.526139W	12,505	1,526	EE	3′ single-stranded overhang	68.7	25
Pondwater	42.389388N, 72.526906W	5,074	619	EE	3′ single-stranded overhang	68.5	25
Yasuo	42.4019N, 72.5231W	12,574	1,544	EE	3′ single-stranded overhang	68.7	25

Double-stranded DNA was extracted from high-titer lysates of each phage using a zinc chloride precipitation method (4), prepared for sequencing using the NEBNext Ultra II kit (New England Biolabs, Ipswich, MA), and sequenced using an Illumina MiSeq instrument (v3 reagents) at the Pittsburgh Bacteriophage Institute. Sequencing provided 150-bp single-end reads with 12,505-fold coverage for Anseraureola, 5,074-fold coverage for Pondwater, and 12,574-fold coverage for Yasuo (Table 1). Raw reads were assembled and quality control checks were performed with Newbler v2.9 and Consed v29.0, respectively (5, 6). Phage termini were identified via read start buildups and similarity to other known phage genome sequences. The results (genome size, GC content, and predicted numbers of genes and termini) are listed in Table 1. Based on gene content similarity of at least 35% to phages in the Actinobacteriophage Database, Anseraureola, Pondwater, and Yasuo were assigned to cluster EE (1, 7). The three genomes were autoannotated using Glimmer v3.02 (8) and GeneMark v2.5 (9), and the annotations were then manually refined using Phamerator (10), DNA Master v5.23.6 (http://phagesdb.org/DNAMaster), and PECAAN. No tRNA genes were identified by ARAGORN v1.2.38 (11) or tRNAscan-SE v2.0 (12). All analyses were conducted using default settings. All three phages have the typical genomic architecture seen in *Microbacterium* cluster EE genomes (1), in which the left arm of the genome contains structural assembly genes, including a major capsid and protease fusion protein that is common in small-genome siphoviruses, and the right arm contains the lysis cassette, putative transcriptional regulators, and an HNH endonuclease.

### Data availability.

Anseraureola findings are available in GenBank with accession no. ON108642 and Sequence Read Archive (SRA) accession no. SRX14443514. Pondwater findings are available in GenBank with accession no. ON081334 and SRA accession no. SRX14483235. Yasuo findings are available in GenBank with accession no. ON108648 and SRA accession no. SRX14485107.
